# Antiviral Potential of Natural Resources against Influenza Virus Infections

**DOI:** 10.3390/v14112452

**Published:** 2022-11-05

**Authors:** Johanna Eichberg, Elena Maiworm, Markus Oberpaul, Volker Czudai-Matwich, Tim Lüddecke, Andreas Vilcinskas, Kornelia Hardes

**Affiliations:** 1 Department of Bioresources, Fraunhofer Institute for Molecular Biology and Applied Ecology, Ohlebergsweg 12, 35392 Giessen, Germany; 2 BMBF Junior Research Group in Infection Research “ASCRIBE”, Ohlebergsweg 12, 35392 Giessen, Germany; 3 LOEWE Centre for Translational Biodiversity Genomics (LOEWE-TBG), Senckenberganlage 25, 60325 Frankfurt, Germany; 4 Institute of Insect Biotechnology, Justus-Liebig-University of Giessen, Heinrich-Buff-Ring 26–32, 35392 Giessen, Germany

**Keywords:** influenza virus, infection, natural products, bio resources, bacteria, extracts, fungi, antimicrobial peptides, antiviral drugs, venom

## Abstract

Influenza is a severe contagious disease caused by influenza A and B viruses. The WHO estimates that annual outbreaks lead to 3–5 million severe infections of which approximately 10% lead to the death of the patient. While vaccination is the cornerstone of prevention, antiviral drugs represent the most important treatment option of acute infections. Only two classes of drugs are currently approved for the treatment of influenza in numerous countries: M2 channel blockers and neuraminidase inhibitors. In some countries, additional compounds such as the recently developed cap-dependent endonuclease inhibitor baloxavir marboxil or the polymerase inhibitor favipiravir are available. However, many of these compounds suffer from poor efficacy, if not applied early after infection. Furthermore, many influenza strains have developed resistances and lost susceptibility to these compounds. As a result, there is an urgent need to develop new anti-influenza drugs against a broad spectrum of subtypes. Natural products have made an important contribution to the development of new lead structures, particularly in the field of infectious diseases. Therefore, this article aims to review the research on the identification of novel lead structures isolated from natural resources suitable to treat influenza infections.

## 1. Introduction

Influenza A (IAV) and B (IBV) viruses cause severe, contagious infections. Typical symptoms include cough, fever, sore throat, and general malaise, which last 1–2 weeks. Particularly high-risk patients, like elderly or immunocompromised people, are at risk of developing serious, life-threatening complications [1]. For example, influenza infection can manifest as primary viral or secondary bacterial pneumonia. Additionally, non-respiratory complications like myositis or encephalomyelitis may occur. Worldwide, these annual epidemics result in an estimated 3–5 million severe disease cases and 300,000–500,000 deaths [2]. Therewith associated costs to healthcare systems and the loss of working hours are a severe socioeconomic burden [3,4].

Annual vaccination is the most important preventive measure against influenza. Influenza viruses mutate quickly due to antigenic drift (conventional mutation) and antigenic shift (re-assortment of the segmented genome), so annual adaptation to the most prevalent virus strains is inevitable and vaccination is required annually for at-risk groups. The World Health Organization (WHO) issues a recommendation to manufacturers in spring for the composition of the vaccine every year [5]. Production is based on embryonated chicken eggs and therefore takes about half a year, so that the vaccine is available in fall. In the event of an influenza pandemic caused by a novel or unexpected strain, the same production time is problematic, because no vaccine is initially available, which was the case for the influenza A (H1N1) 2009 pandemic [6].

Effective anti-influenza drugs are urgently needed to address the limitations of the current influenza vaccination program. Thus far, only two classes of anti-influenza drugs have been approved worldwide, although additional drugs are available in certain countries. Most of these compounds suffer from a very limited efficacy and the rapid emergence of resistance. Therefore, it is imperative to steadily explore additional options for the treatment or prevention of influenza, particularly the vast range of natural products synthesized by microbes and animals. Starting from the structure and replication cycle of the influenza virus, here we provide an overview of the approved drugs and then discuss the therapeutic potential of natural products that inhibit influenza virus replication.

### 1.1. Biology of Influenza Viruses

IAVs have been found in a wide range of mammals and birds, including humans, swine, and poultry, all seemingly derived from a viral ancestor in wild birds [2]. Therefore, the natural reservoir for IAV is waterfowl like ducks, geese, or wild aquatic birds [7,8]. In contrast, the so-called bat influenza-like viruses might be an exception because their origin is still unknown [2]. IBV occurs predominantly in humans and is not yet known to have an animal reservoir. The closely related influenza C viruses cause only mild or even asymptomatic infections and in case of influenza D viruses no human infections have been observed.

**Figure 1:**
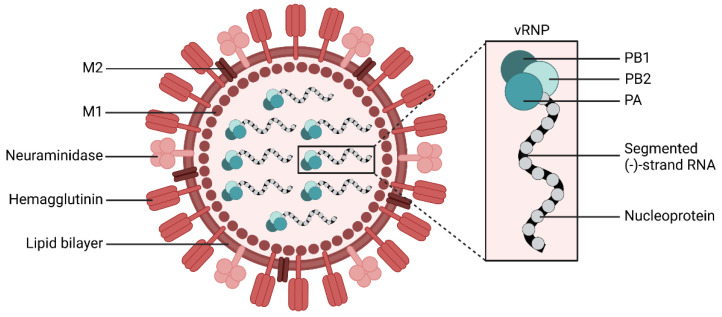
Schematic representation of an influenza A virus. The enveloped virus contains eight single stranded RNA segments encoding for each viral protein and are bound to nucleoproteins. Together with the viral polymerase, they form the viral ribonucleoprotein (vRNP). The viral RNA-dependent RNA polymerase is a heterotrimer consisting of the polymerase acid (PA), polymerase basic 1 (PB1), and polymerase basic 2 protein (PB2). The surface glycoproteins hemagglutinin (HA) and neuraminidase (NA) mediate viral entry and viral release, respectively. The matrix protein (M1) forms a coat inside the virus envelope. The membrane protein (M2) is a proton ion channel, which mediates the acidification of the endosomally entrapped virus. Created with BioRender.com (accessed on 20 September 2022).

Influenza viruses have a negative sense single strand RNA genome and belong to the family *Orthomyxiviridae*. The viral genome of IAV and IBV consists of eight RNA segments, which encode the virus surface glycoproteins hemagglutinin (HA) and neuraminidase (NA), the nucleoprotein (NP), the virus polymerase subunits PA, PB1, and PB2, the matrix protein M1, the ion channel M2 as well as the nonstructural proteins NS1 and the so called nuclear export protein NS2 (see Figure 1 for a schematic representation of an IAV) [9].

**Figure 2:**
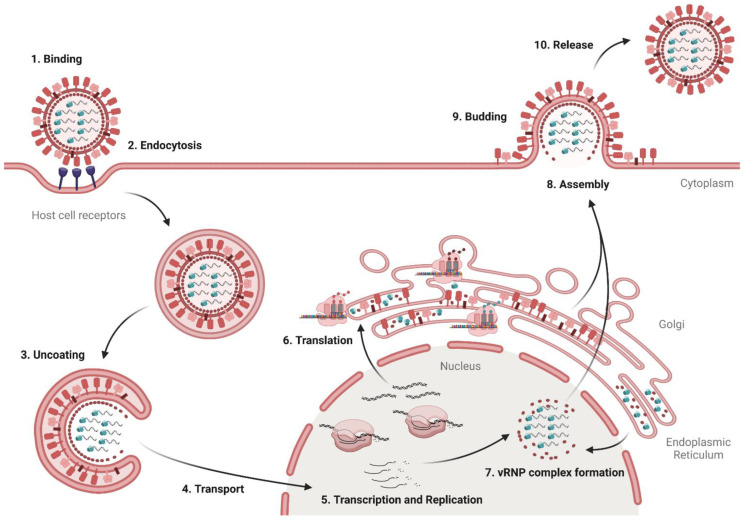
Replication cycle of influenza virus. First, the virus binds via HA to sialic acid residues on the host cell (Step 1) and is taken up into the cell via endocytosis (Step 2). After acidification of the endosome and viral interior, the vRNPs are released in the cytosol (Step 3) and transported in the nucleus (Step 4), where the viral RNA is transcribed and replicated (Step 5). Viral mRNAs are transported in the cytosol for translation (Step 6). NPs and the subunits of the viral polymerase PA, PB1, and PB2 are transported back in the nucleus to form new vRNP complexes (Step 7). vRNPs and HA, NA, and M1 are transported to the cell surface for assembly (Step 8). The virion buds from the cell surface (Step 9). In the final step, NA cleaves sialic acids from the cell surface and progeny virions, enabling virus release from infected cells (Step 10). Adapted from “HIV Replication Cycle”, by BioRender.com (2022) (accessed on 20 September 2022). Retrieved from https://app.biorender.com/biorender-templates (accessed on 20 September 2022).

The replication cycle of influenza viruses can be divided into ten steps (see Figure 2). At the beginning of the replication, the virus binds to sialyloligosaccharides on glycoproteins or lipids on the surface of bronchial host epithelial cells via its surface protein HA [10]. The low affinity of HA for the receptors with a dissociation constant K_D_ value > 0.1 mM is compensated by the simultaneous formation of multiple bonds [11]. Subsequently, receptor-mediated endocytosis facilitates the absorption of the virus into the cell. As the endosome matures, the luminal pH decreases due to V-ATPase activity, which results in the import of protons [12]. The low pH causes HA to undergo an irreversible conformational change [13]. A prerequisite for this conformational change is the preceding cleavage of the precursor HA0 into the subunits HA1 and HA2, which is mediated by host proteases such as TMPRSS2 [14]. By forming a hairpin-like conformation of the fusion peptides, lipids from the endosome and viral membranes are bound together and a fusion pore is formed [10,15]. Protons enter the viral capsid via M2 channels, detaching the vRNP complexes from the M1 matrix protein [16]. The eight negative strand RNA segments and associated proteins enter the cytosol through the fusion pore, and are subsequently transported to the nucleus by importins [17].

In the nucleus, the viral RNA is transcribed into mRNA and replicated to form positive strands, which serve as templates for the synthesis of new viral negative strand RNA by the viral RNA polymerases [11]. The mRNA is transported into the cytosol and translated at the rough endoplasmic reticulum (ER). The coat proteins HA and NA are transported to the cell surface through the rough ER and the Golgi apparatus. Along this pathway, they undergo posttranslational modification and are assembled into the typical trimers and tetramers, respectively. Proteins with nuclear transport signals migrate to the nucleus, where they assemble into the vRNP complexes. The newly-formed viral proteins and the negative strand RNA accumulate at the cell surface, where a new virus forms by budding. The virus is still bound to the terminal sialic acid of the host cell receptors by HA. Subsequently, NA cleaves the sialic acid from the host cell glycoproteins and the weakly-bound sialic acid residues rapidly diffuse from the newly-formed virus [10].

### 1.2. Antiviral Therapy of Influenza Infections

**Figure 3:**
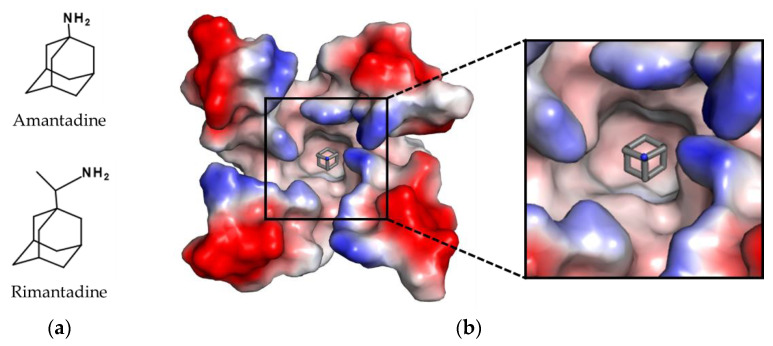
Structures of approved M2 channel inhibitors. (**a**) Chemical structure of amantadine and rimantadine. (**b**) Complex of amantadine (shown as sticks with grey carbon atoms and nitrogen in blue) in the pore of the M2 channel. The surface of the M2 channel was colored according to is calculated negative (−64 kT/e, red) and positive (+64 kT/e, blue) electrostatic potential. The structure was determined by X-ray crystallography (10.2210/pdb3C9J/pdb) [23]. The figure was generated with PyMOL Molecular Graphics System Version 2.0 (Schrödinger, LLC, New York, NY, USA).

Based on the replication cycle of the influenza virus (see Figure 2), there are numerous, potential targets for antiviral therapies. However, in most countries only two different drug classes are currently approved for the treatment of influenza infections: M2 ion channel and NA inhibitors. The first drugs approved for the treatment of influenza infections were amantadine in 1966 and rimantadine in 1994 (see Figure 3). Both compounds target the matrix protein M2 of IAV, which functions as a proton channel in the viral envelope. During replication the M2 channel transports protons from the acidic endosomal environment into the virus lumen. As a result, the decrease of pH leads to the dissociation of the vRNP-M1 complexes, so that the vRNPs are released for transport into the nucleus. Inhibition of acidification effectively prevents viral uncoating and subsequent release of vRNPs. However, M2 blockers suffer from three major drawbacks: (1) due to structural differences in the proton channel, M2 blockers are only effective against IAV, but ineffective against IBV. (2) In order to suppress viral replication, they must be taken shortly after infection. (3) High prevalence of resistance: The first reports on mutations related to resistances against amantadine were published in the 1980s [18]. However, prevalence of these mutations remained below 1% until the early 2000s [19]. This changed drastically during the season 2004/2005, when resistant H3N2 isolates began to spread worldwide, reaching almost 100% prevalence in the following season [20,21]. As single mutations in M2 (like S31N or L26F) are sufficient for resistance development against the aminoadamantanes, both compounds are no longer recommended for the treatment of influenza infections [22].

**Figure 4:**
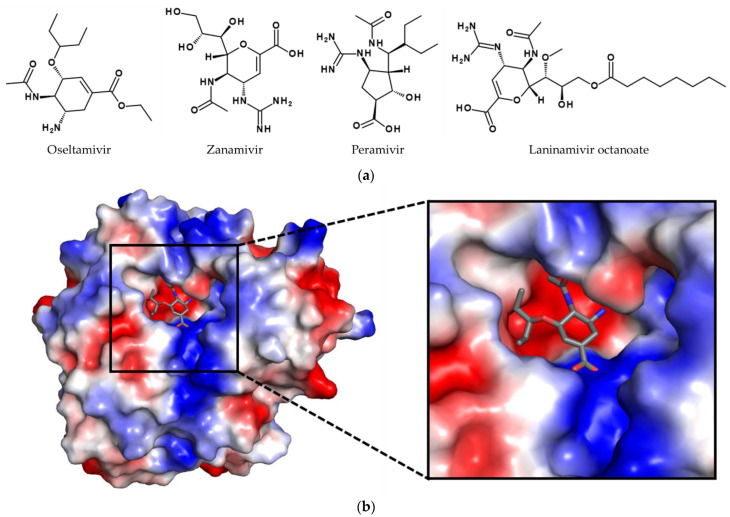
Structures of approved NA inhibitors. (**a**) Structures of oseltamivir, zanamivir, peramivir, and laninamivir octanoate. (**b**) Crystal structure of oseltamivir (shown as sticks with grey carbon atoms, oxygen in red, and nitrogen in blue) in the binding cleft of a N1 NA. The surface of the NA was colored according to is calculated negative (−58 kT/e, red) and positive (+58 kT/e, blue) electrostatic potential. The structure was determined by X-ray crystallography (10.2210/pdb2HU0/pdb) [38]. The figure was generated with PyMOL Molecular Graphics System Version 2.0 (Schrödinger, LLC, New York, NY, USA).

Due to this, NA inhibitors are currently the most important drugs for antiviral therapy, including oseltamivir, zanamivir, laninamivir, and the synthetic drug peramivir, mimicking a natural product [24,25] (see Figure 4). The target of these compounds is the NA on the viral surface. NA is involved in several steps during infection [26,27]: (1) It enhances the viral transport through the mucus. (2) If the virus becomes entrapped by binding to sialic acid residues of non-receptor molecules, the NA removes the unwanted residues. (3) It facilitates the movement of the virus on the cell surface to find active spots for endosomal uptake. (4) After budding, the neuraminidase releases the progeny virions from the cell surface by cleaving the sialic acid residues of cell surface glycoconjugates. (5) It removes sialic acid residues from the viral surface to prevent aggregation of the virions.

Regarding their development, the first inhibitors were already synthesized in the 1960s [28]. Especially the progress in the field of X-ray crystallography enabled the design of specific and potent inhibitors of the influenza virus NA: i.e., zanamivir was developed based on the crystal structures of NA in complex with sialic acid [29]. However, zanamivir suffers from poor bioavailability and fast elimination [30,31]. To address this problem, a prodrug strategy was applied. For example, oseltamivir is administered as ethyl ester and subsequently converted to its active form oseltamivir carboxylate by esterases in the intestinal tract and liver [19]. In contrast to zanamivir and oseltamivir, peramivir has a cyclopentane scaffold. Additionally, laninamivir octanoate was developed. It is a structural analog of zanamivir and the only long-acting NA inhibitor available.

Regarding resistances, the first cases of oseltamivir resistance were reported in the season 2007/2008. However, the number differs strongly from season to season. During the season 2008/2009 in some parts of the world, oseltamivir resistance was observed in more than 90% of infections caused by seasonal H1N1 strains [32]. In contrast, at the beginning of the influenza A (H1N1) 2009 pandemic, only 1.5% of clinical isolates were resistant against the drug [33]. Responsible for the resistance development is in many cases the H274Y (depending on the amino acid numbering also known as H275Y) mutation. This mutation prevents the conformational change needed for oseltamivir binding and thereby drastically reduces the susceptibility of the NA towards oseltamivir [33,34]. Due to a similar binding mode, peramivir is also affected by this mutation. In contrast, binding of zanamivir does not require any structural changes, so viruses harboring 274Y remain sensitive [35]. Besides H274Y, other mutations like R292K or N294S have been reported, also affecting the susceptibility toward the NA inhibitors [35,36].

In the last years, the efficacy of NA inhibitors has been debated. For optimal efficacy, the drug has to be administered within 36 h twice daily after the onset of symptoms. Then, the duration of illness can be reduced by 30% with a 38% reduction in severity of symptoms as well as lowering the risk of developing secondary complications [37].

In some countries additional compounds are approved, like the recently developed cap-dependent endonuclease inhibitor baloxavir marboxil, polymerase inhibitor favipiravir, or umifenovir.

**Figure 5:**
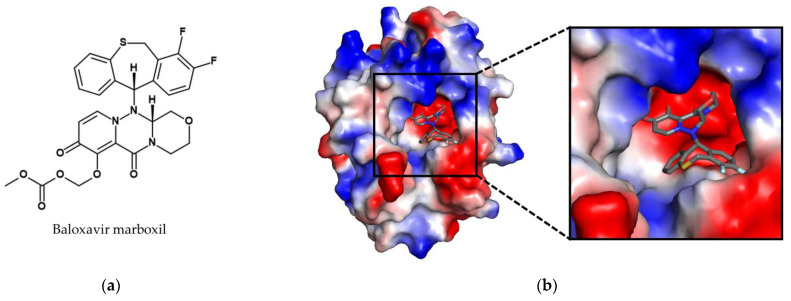
Structure of the approved cap-dependent endonuclease inhibitor baloxavir marboxil. (**a**) Chemical structure of the prodrug baloxavir marboxil. (**b**) Crystal structure of baloxavir (shown as sticks with grey carbon atoms, oxygen in red, nitrogen in blue, and fluorine in cyan) in the binding cleft of endonuclease. The surface of the endonuclease was colored according to is calculated negative (−69 kT/e, red) and positive (+69 kT/e, blue) electrostatic potential. The structure was determined by X-ray crystallography (10.2210/pdb6FS6/pdb) [40]. The figure was generated with PyMOL Molecular Graphics System Version 2.0 (Schrödinger, LLC, New York, NY, USA).

In 2018, the synthetically-derived small molecule inhibitor baloxavir marboxil was approved for the treatment of acute uncomplicated influenza infections in both Japan and the United States, followed by other countries such as Australia and in the European Union (see Figure 5). It is the first new anti-influenza drug with a novel mode of action approved in almost 20 years. Baloxavir marboxil is an orally available prodrug that is converted to its active metabolite baloxavir by hydrolysis. The target of baloxavir is the acidic polymerase protein PA, which together with PB1 and PB2 forms the viral RNA polymerase complex [39]. The endonuclease activity of PA is responsible for host mRNA cap-snatching, a process required for the generation of the 5′ capped oligonucleotides which serve as primers for viral mRNA synthesis. This step is a prerequisite for influenza virus mRNA production and its inhibition halts virus replication. Unlike most other influenza drugs, it is administered only in a single dose, which is an advantage for therapy in terms of compliance.

A study on healthy individuals with confirmed IAV and IBV infections treated with baloxavir marboxil found that 9.7% of isolates acquired the I38X mutation in PA [41]. The occurrence of this mutation was associated with higher viral load as well as prolonged viral detection and symptoms compared with individuals without the mutation.

Favipiravir is an inhibitor of the RNA-dependent RNA polymerase [42]. Like baloxavir marboxil, it is a prodrug and undergoes ribosylation and phosphorylation after oral administration. During replication, favipiravir ribofuranosylphosphate acts as nucleotide analog and is incorporated into the viral RNA, leading to lethal mutagenesis and by that inhibits replication [19]. Favipiravir demonstrated safety in humans and efficacy against influenza infections in phase II and phase III clinical trials. However, the authors of a phase III clinical trial concluded inconsistent illness alleviation in uncomplicated influenza. Studies with higher doses and antiviral combinations for treating serious influenza are highly recommended and seen as warranted [43]. However, favipiravir is probably teratogenic in animal studies, and larger trials in humans are necessary [44,45]. So far, it is limited to pandemic preparedness and not for use against regular seasonal flu, additionally, its production condition is limited to Japan. Besides influenza, it also inhibits the replication of other RNA viruses like Rift Valley fever virus, West Nile virus, or yellow fever virus [42]. Furthermore, during the recent SARS-CoV-2 pandemic or the Ebola outbreak of 2014 it was tested against these pathogens [46,47]. Favipiravir showed a promising protective effect against Ebola virus in laboratory-infected mice [48]. In clinical trials during the outbreak, a trend for improved survival rates in favipiravir-treated patients was observed. However, a clear conclusion on its efficacy could not be made [49,50,51]. The data situation is comparable for SARS-CoV-2: a slight beneficial effect was observed in some studies [52,53,54]. However, due to limitations like small sample size or combination of favipiravir with additional drugs, no clear conclusion can be drawn either.

Similar to favipiravir, umifenovir was developed for the treatment of IAV and IBV infections. It inhibits the virus entry and stimulates the immune response of the cell [55]. It was also further tested against a broad panel of different viruses and showed activity against several other viral pathogens like Zika virus, West Nile virus, and tick-borne encephalitis virus [56].

In summary, the number of drugs available for the treatment of influenza is very limited, and their long-term efficacy is severely hampered by the rapid development of resistant strains. This highlights the urgent need for the discovery and development of new antiviral therapeutics with the broadest possible spectrum of activity against different strains.

## 2. Natural Resources in Drug Development

Natural products have been used since ancient times as traditional medicine to cure diseases [57]. Their use already provided the first knowledge about their application, but also about their effectiveness and safety. The discovery of natural products in the modern sense became possible with the advances in isolation and purification techniques and the possibility of studying the effects of substances and extracts on model systems beginning in the 19th century [58]. Since then, a plethora of extracts and individual compounds have been isolated from various sources such as plants, bacteria, fungi, and animals for drug discovery [59,60,61,62,63,64,65]. These compounds are characterized by an enormous variety of scaffolds and structures that often do not conform to the classical molecular properties used for small molecule design, such as Lipinski’s rule of five [66]. However, natural products often serve as lead structures for optimization [67]. This has been used to design several drugs such as the semi-synthetic penicillin V or the chemotherapeutic paclitaxel.

Natural products have played a central role in the development of novel anti-infectives. However, most of the research was conducted in the field of antibiotics. Nevertheless, several hundred compounds have also been tested for their antiviral activity [68]. Especially, compounds derived from plants and herbs have been extensively studied. For example, polyphenols from *Geranium sanguineum* [69], flavonoids such as gingetin isolated form *Gingko biloba* [70] or lignans, such as the rhinacanthin E and F extracted from *Rhinacanthus nasutus* [71] show an activity against influenza viruses. However, from 1981 to 2019 neither an antibacterial nor an antiviral drug of botanical origin has been approved by the FDA [25,67].

Since there are many excellent review articles (e.g., [61,62,63,64,65]) which focus on the antiviral potential of compounds derived from plants against influenza, we put the focus of this review on compounds and extracts derived from fungi and bacteria, as well as toxins and antimicrobial peptides (AMPs) derived from animals. A table listing all compounds reviewed can be found as supplement.

## 3. Toxins and Defensive Peptides from Animals Tested for Antiviral Properties

Many animals have evolved venoms to protect themselves from predators or to capture prey [72]. These venoms consist of various proteins, peptides, and small molecules, but the majority of toxic components are peptides [73]. Other animals, including some amphibians, lack a delivery system for venoms and are therefore classified as poisonous animals. Nevertheless, they secrete a variety of small molecules and peptides with diverse antimicrobial, immunomodulatory, and regulatory activities that constitute an effective defensive system [74].

The toxins found in animal venoms and poisons often target the nervous or cardiovascular system, but many also possess potent antimicrobial and/or antiviral properties, which might be suitable for therapeutic applications [75]. Such components have therefore been examined for the development of new drugs against influenza [76]. For example, alloferons, defense peptides isolated from bacteria-challenged larvae of the blow fly *Calliphora vicina*, exhibited antiviral activity in NMRI mice challenged with influenza strains A/Aichi/2/68 (H3N2) and B/Lee/1/40 [77]. The cationic peptide mucroporin (LFGLIPSLIGGLVSAFK) derived from the scorpion *Lychas mucronatus* has antibacterial properties [78]. Its derivative mucroporin-M1 (LFRLIKSLIKRLVSAFK) was designed by substitution of several glycine residues and one proline by the basic amino acids arginine and lysine. It showed antiviral activity against an influenza H5N1 pseudovirus based on human cytomegalovirus modified with the HA of wild-type H5N1 and NA from A/Puerto Rico/8/34 with an EC_50_ value of 1.03 µM (a definition for the values given can be found in the nomenclature section) [79]. However, these antiviral effects could not be demonstrated for the natural peptide mucroporin. The peptide melittin, isolated from bee venom, can inhibit the infectivity of influenza strain A/Puerto Rico/8/34 (H1N1) in vitro with an EC_50_ value of 0.40 µM [80]. This peptide was also shown to inhibit influenza A subtype H1N1 infection in mice. Similarly, the host defense peptide urumin, isolated from the skin of the Indian frog *Hydrophlax bahuvistara*, targets the stalk region of HA H1 and has antiviral effects in MDCK cells with an IC_50_ of 3.8 µM. Additionally, it protected mice against H1N1 influenza A/Puerto Rico/8/34, resulting in the survival of 70% of the infected mice compared to 20% of the untreated controls [81]. Isolated from banded krait (*Bungarus fasciatus*) venom, the peptide BF-30 is another promising candidate for influenza treatment [82]. Xu et al. explored its antiviral activity against influenza A/Beijing/32/92 (H3N2), A/FM/1/47 (H1N1), and an oseltamivir-resistant mutant strain (H275Y, H1N1) and determined EC_50_ values of 7.4, 5.2, and 18.9 µM, respectively. Furthermore, in vivo experiments conducted in mice showed 50% survival at a dosage of 4 µM with a 2-log reduction of viral titers in the lungs. The authors suggest that the antiviral effect of BF-30 occurs during attachment and permeation phase by causing membrane fusion of virus particles, thereby inhibiting virus infections. As BF-30 does not interact with HA and NA, both being prone to becoming drug resistant by acquiring adaptive mutations, it is an interesting candidate, which should be further examined for therapeutic application.

Overall, the above work highlights that animal venoms and poisons are an interesting source for the discovery of novel lead structures for the treatment of influenza and likely other viruses as well. However, due to methodological limitations and anthropocentric bias towards larger and medically significant species, only a minor fraction of animal venom and poison systems have been explored and most biomolecules remain unknown to date [83,84]. The recent developments of novel systems biology and biotechnology platforms allow the study of venoms and poison of virtually all species within the animal kingdom and thus the discovery of many additional compounds with anti-influenza activity can be expected [85].

## 4. Fungal Extracts and Compounds with Anti-Influenza Properties

The antimicrobial activity of fungi is well-documented [86] and has been recently reviewed [87]. The majority of the extracts and compounds isolated were tested as antibiotics in bacterial infection models. However, a few extracts, e.g., those from *Basidiomycetes*, were examined for their antiviral activities, including influenza viruses [88]; their potential was reviewed earlier [89].

### 4.1. Terrestrial Fungi

As mentioned above, Krupodorova et al. assessed antiviral activity of extracts from *basidiomycetes* mycelium, cultivated on natural substrate [88]. They tested mycelial extracts from *Auriporia aurea*, *Fomes fomentarius*, *Pleurotus ostreatus*, *Lyophyllum shimeji*, *Lentinus edodes*, *Pleurotus eryngii*, *Flammulina velutipes*, *Ganoderma lucidum*, *Schizophyllum commune*, and *Trametes versicolor*. All extracts were able to inhibit replication of A/FM/1/47 (H1N1), with *G. lucidum* and *T. versicolor* being the most potent extracts (EC_50_ values of 0.077 mg/mL for both extracts).

The fungal compound cyclosporin A (CsA) produced by *Tolypocladium inflatum* has been shown to inhibit virus replication in vitro. Over the past decade, several groups tried to elucidate its therapeutic potential using a range of different viral strains and target cells. For example, in a study by Liu et al., the level of M1 protein was reduced in CsA-treated HEK 293T cells infected with influenza virus A/WSN/33 (H1N1) [90]. Similarly, Hamamoto and colleagues reported IC_50_ values of 1.45 and 1.78 µM in CsA-treated A549 and MDCK cells, respectively, infected with influenza virus A/Puerto Rico/8/34 (H1N1) [91]. More recently, the antiviral activity of CsA was demonstrated against a broad panel of IAV and IBV strains in MDCK cells, with EC_50_ values of 2.08, 2.60, 0.37, 11.68, 2.25, 3.16, and 0.97 µM against strains A/WSN/33 (H1N1), A/Udorn/72 (H3N2), A/Switzerland/9715293/13 (H3N2), A/California/07/2009 (H1N1), A/Texas/04/2009 (H1N1), B/Brisbane/60/08 (Victoria lineage), and B/Phuket/3073/13 (Yamagata lineage), respectively [92]. Although the mechanism of action is still unclear, CsA is known to target the posttranscriptional steps of influenza virus replication in a Cyclophilin A (CypA)-independent manner. Two other compounds isolated by solid-state fermentation of *Stachybotrys* sp. RF-7260 also showed antiviral activity [93]. Stachyflin and acetylstachyflin were tested against influenza A/WSN/33 (H1N1) in MDCK cells and inhibited virus replication with IC_50_ values of 0.003 µM and 0.23 µM, respectively. Yoshimoto et al. found evidence that stachyflin interfered with HA conformational change and therefore inhibited HA-mediated virus-cell fusion [94].

Produced by different species of the genus *Chaetomium*, aureonitol represents yet another interesting metabolite against influenza virus [95]. The tetrahydrofuran derivate was obtained from *Chaetomium coarcatatum* and showed antiviral activity against influenza A (H3N2) with an EC_50_ of 0.1 µM in MDCK cells. Aureonitol inhibited influenza hemagglutination and, consequently, impaired virus adsorption significantly.

Awadh et al. tested ethanolic extracts of fruit bodies (EE^a^) and mycelial cultures (EE^b^) from *Inonotus hispidus* against influenza viruses A/Brazil/11/78 (H1N1), A/Hongkong/1/68 (H3N2) and influenza B/Singapore/222/79 [96]. At 80 µg/mL both EE^a^ and EE^b^ showed antiviral activity in form of viral titer reduction in allantois against both IAV and IBV strains. Hispidin and hispolon, two compounds isolated from the ethanolic extracts, even reduced the viral titer of influenza virus A and B at 40 µg/mL. RC-183, a protein purified from the edible mushroom *Rozites daperata* also exhibits antiviral activity against a broad spectrum of viruses including A/Shanghai (H3N2) [97].

Statins are HMG-CoA (3-hydroxy-3-methyl-glutaryl-coenzyme A) reductase inhibitors and the most common hypercholesterolemia drugs due to their ability to decrease low-density lipoprotein (LDL) cholesterol levels [98]. Statins from the fungus *Penicillium citrinum* also display pleiotropic effects and are classed as immunomodulators [99]. Potential activity against influenza virus was observed in clinical studies, during which statin-treated patients showed a lower risk of influenza and fatal COPD [100]. Other studies produced controversial results. For example, statins were found to remarkably reduce the mortality rate in hospitalized patients [101], whereas in another study they were co-administered with methylprednisolone and activated protein C, thus it remains unclear which drug was responsible for the observed effect [102]. In addition, several studies do not report about any positive effects of statins in influenza patients. This ambivalence of reports and observations is probably due to the range of different methods involved but clearly reflects difficulties in interpreting clinical data [103,104]. For example, fluvastatin was shown to reduce the intracellular level of viral nucleoprotein mRNA in MDCK cells infected with influenza virus A/Guangdong/3/2009 (H1N1), with an IC_50_ of 10.45 µM, but when administered alone this drug was highly cytotoxic [105]. Treatment with simvastatin did not reduce morbidity, mortality, or viral load in BALB/c mice infected with influenza virus A/Chicken/Korea/Gimje/2008 (H1N1) or A/Mexico/4482/2009 (pH1N1) [106]. Similar results were observed in C57BL/6 mice treated with rosuvastatin, which did not influence viral clearance, weight loss or mortality caused by strains A/Udorn/72 (H3N2) or A/WSN/33 (H1N1) [107]. Given that the antiviral effect of statins could not be confirmed in vitro or in vivo, the pleiotropic effects of statins may modulate the immune system and therefore influence the outcome of an influenza infection in a positive way.

**Figure 6:**
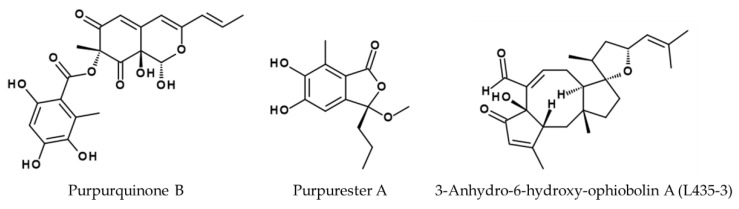
Structures of purpurquinone B [108], purpurester A [108], and 3-anhydro-6-hydroxy-ophiobolin A (L435-3) [114] derived from fungi with high in vitro and in vivo efficacy against A/WSN/33 infection.

Two other *Penicillium* species also secrete bioactive compounds with antiviral activity against influenza. Purpurquinone B (see Figure 6), purpurquinone C, purpurester A (see Figure 6), and TAN-931 are produced by *Penicillium purpurogenum* JS03-21, and are active against influenza virus A/Puerto Rico/8/34 (H1N1) with IC_50_ values of 61.3, 64.0, 85.3, and 58.6 µM, respectively [108]. Sorbicatechol A and B isolated from *Penicillium chrysogenum* PJX-17 also showed antiviral activity against an unspecified H1N1 influenza A strain with IC_50_ values of 85 and 113 µM, respectively [109]. *Trichoderma atroviride* FKI-3849 produces wickerol A, which showed antiviral properties against influenza A/Puerto Rico/8/34 (H1N1) with an IC_50_ value of 0.24 µM in MDCK cells [110]. The same research group also isolated herquline A from culture broth of *Penicillium herquei* FKI-7215. Herquline A showed antiviral activity against influenza A/Puerto Rico/8/34 (H1N1) with an IC_50_ value of 30.80 µM in MDCK cells [111]. Isolated from a sponge in the genus *Amphimedon*, the microbial strain *Truncatella angustata* XSB-01-43 produces truncateol O, which showed antiviral activity against A/WSN/33 (H1N1) with an IC_50_ value of 30.4 µM in 293T cells [112].

The phytopathogenic fungus *Bipolaris oryzae* is a source of ophiobolin derivatives that inhibit the proliferation of cancer cell lines and show antimicrobial activity against methicillin-resistant *Staphylococcus aureus* [113]. Furthermore, 3-anhydro-6-hydroxy-ophiobolin A (L435-3) was active against influenza virus in vitro and in vivo (see Figure 6) as demonstrated by the same group. When tested for its antiviral potential in A549 cells, MDCK cells, and in BALB/c mice infected with the mouse-adapted influenza virus strain A/WSN/33 (H1N1), strong antiviral effects could be observed [114]. The IC_50_ of L435-3 against the WSN strain in MDCK cells was 0.365 µM, while the treatment of infected A549 cells with 0.5 µM L435-3 caused a strong reduction in the virus titer in HA and plaque assays. Consistent with these findings, the administration of L435-3 to mice resulted in less severe symptoms and weight loss. Moreover, viral titers in the lungs of WSN-infected mice were reduced by L435-3, suggesting that viral replication was impaired in vitro and in vivo. Microarray analysis and real-time PCR confirmed the induction of type III interferon gene expression, leading to an antiviral state associated with the L435-3 mechanism of action, although the precise mechanism and other potential protective effects need further investigation.

Originally identified as an antibiotic against *Bacillus anthracis*, mycophenolic acid (MPA) and particularly its modified compounds are now used as immunosuppressant drugs [115]. MPA, isolated from *Penicillium brevicompactum* inhibits the inosine monophosphate dehydrogenase (IMPDH), which is the rate-controlling enzyme for GMP biosynthesis [116]. Chan et al. investigated the antiviral property of MPA against influenza A/WSN/33 (H1N1) and determined an IC_50_ value of 0.24 µM in MDCK cells [117]. They further tested MPA against clinical isolates A/Hong Kong/415742/2009 (H1N1), A/Hong Kong/402467/2014 (H1N1), A/Hong Kong/447572/2011 (H3N2), A/Hong Kong/460611/2013, A/Anhui/1/2013 (H7N9), A/Zhejiang/DTID-ZJU01/2013 (H7N9) as well as B/Hong Kong/411989/2011, and Hong Kong/446321/2013 [118]. In a cell viability assay using MDCK cells, MPA inhibited replication of IAV and IBV with IC_50_ values of 1.510 µM for A/H1N1/415 and 0.208 µM for B/411, respectively. Addition of guanosine reverted the antiviral effect of MPA; therefore, the authors conclude that the inhibition of virus replication by MPA is caused by host-cell guanosine depletion. Since the prodrug of MPA, mycophenolate mofetil, has already been approved for clinical use, MPA represents an interesting candidate regarding treatment of influenza infections.

Another immunomodulator is myriocin. It has been isolated from *Isaria sinclairii* and was investigated regarding its antiviral property. Myriocin inhibits serine palmitoyltransferase and therefore impairs sphingolipid biosynthesis [119]. Tafesse et al. found that the display of viral glycoproteins is affected when sphingolipid biosynthesis is compromised due to a trafficking defect from HA and NA from the tans-Golgi network to the cell surface [120]. Fumonisin B1 (FB1), produced by several *Fusarium* species, also suppresses sphingolipid biosynthesis. It showed antiviral activity against A/Puerto Rico/8/34 (H1N1), A/Memphis/1/71 (H3N2), and B/Lee/40 in MDCK cells [121]. The authors suppose that the mode of action is the reduction of cellular sphingolipids which might affect the trafficking of viral proteins or the assembly of viral proteins at the cell surface.

### 4.2. Marine and Limnic Fungi

Besides terrestrial fungi, marine species living within or on algae, corals, sponges, or other fungi [122] also offer a rich source of new therapeutic compounds [123]. For example, the fungus *Cladosporium sphaerospermum* produces seven compounds with activity against a not further-specified H1N1 influenza [124,125]. Xoglyantryphine, norquinadoline A, desoxynortryptoquivaline, desoxytryptoquivaline, tryptoquivaline, quinadoline B, and cladosin C inhibited H1N1 influenza with IC_50_ values of 85, 82, 87, 85, 89, 82, and 276 µM, respectively. From mangrove sediment *Penicillium camemberti* was also isolated. It produces a variety of compounds, which were tested against IAV (H1N1, not further specified) in MDCK cells. Compounds 3-deoxo-4b-deoxypaxilline, DCA, DPT, 9,10-diisopentenylpaxilline, TTD, emindole SB, 21-isopentenylpaxilline, paspaline, and paxilline showed the following IC_50_ values: 28.3 µM, 38.9 µM, 32.3 µM, 73.3 µM, 34.1 µM, 26.2 µM, 6.6 µM, 77.9 µM, and 17.7 µM, respectively [126].

**Figure 7:**
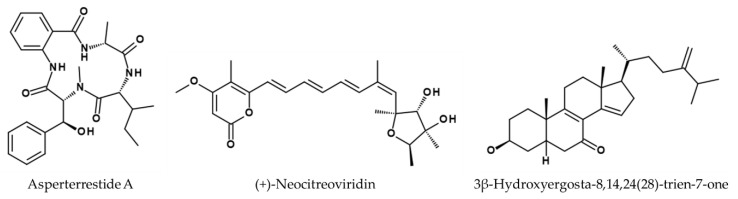
Structures of asperterrestide A [129], (+)-neocitreoviridin [131], and 3β-hydroxyergosta-8,14,24(28)-trien-7-one [127].

*Aspergillus sydowii* ZSDS1-F6 produces the compounds (*Z*)-5-(hydroxymethyl)-2-(6′)-methylhept-2′-en-2′-yl)-phenol, dioricinol, and cordyol C, which showed activity against an H3N2 influenza strain not further specified with IC_50_ values of 57.4, 66.5, and 78.5 µM, respectively [127]. Rubrolide S from *Aspergillus terreus* OUCMDZ-1925 showed activity against A/Puerto Rico/8/34 (H1N1) in MDCK cells with an IC_50_ value of 87.1 µM [128]. Two other *A. terreus* strains have also shown antiviral activity. *A. terreus* SCSGAF0162 produces asperterrestide A (see Figure 7), which was tested against strains A/WSN/33 (H1N1) and A/Hong Kong/8/68 and displayed protective effects, with IC_50_ values of 15 and 8.1 µM, respectively [129]. *A. terreus* Gwq-48 produces the three compounds isoaspulvinone E (IC_50_ = 109.02 µM), aspulvinone E (IC_50_ = 192.05 µM), and pulvic acid (IC_50_ = 94.39 µM) with antiviral activity against A/Puerto Rico/8/34 (H1N1) in MDCK cells [130].

The endophytic fungus *Emericella* sp. HK-ZJ produces multiple isoindolone derivatives, including emerimidine A and B with the ability to inhibit H1N1 influenza virus with IC_50_ values of 201.10 and 296.61 µM, respectively [132].

Isolated compounds from the marine strain *Penicillium* sp. IMB 17-046 exhibited a broad-spectrum antiviral activity including influenza virus A/WSN/33 (H1N1) in HEK 293T-Gluc cells, expressing luciferase as reporter for screening. Three compounds were found to have inhibitory effects: trypolepyrazinol (IC_50_ = 20.4 µM), (+)-neocitreoviridin (IC_50_ = 3.6 µM) (see Figure 7), and 3β-hydroxyergosta-8,14,24(28)-trien-7-one (IC_50_ = 0.5 µM) (see Figure 7) [131]. Spirostaphylotrichin X produced by *Choliobolus lunatus* SCSIO41401, inhibited replication of A/Puerto Rico/8/34 (H1N1) and A/Aichi/2/68 (H3N2) in MDCK cells with IC_50_ values of 1.6 µM and 4.1 µM, respectively [133]. Lou et al. found four antiviral active polyketides produced by *Diaporthe* sp. SCSIO41011, 41012, 41013, and 41014 tested in MDCK cells [134]. Pestalotiopsone B showed antiviral activity against A/Puerto Rico/8/34 (H1N1) (IC_50_ = 2.56 µM) as well as A/Aichi/2/68 (H3N2) (IC_50_ = 6.76 µM). Pestalotiopsone F also was protective against the same strains with IC_50_ values of 21.8 µM and 6.17 µM, respectively. Additionally, the compound called DMXC also inhibited the influenza strains with IC_50_ values of 9.4 µM (for H1N1) and 5.12 µM (for H3N2). Lastly, 5-chloroisorotiorin also exhibited antiviral activities against H1N1 (IC_50_ = 5.12 µM) and H3N2 (IC_50_ = 10.1 µM).

The deep-sea fungus *Eurotium rubrum* F33 (MCCC 3A00287) was isolated from a depth of 2067 m under the South Atlantic Ocean. It produces many antiviral prenylated indole diketopiperazine alkaloid derivatives, but only neoechinulin B showed activity against influenza A/WSN/33 (H1N1) (IC_50_ = 27.4 µM) without exhibiting cytotoxicity. Neoechinulin B targets HA and prevents its interaction with the sialic acid receptor, inhibiting the early stages of virus replication [135]. Furthermore, *Eurotium chevalieri* MUT 2316, isolated from a marine sponge, produces three additional compounds with activity against influenza virus A/Puerto Rico/8/34 (H1N1): dihydroauroglaucin (83.3 µM), physcion (88.0 µM), and neoechinulin D (127.8 µM) inhibited the virus in plaque assays by 100%, 80%, and 70%, respectively, without affecting cell viability (threshold of 70%) [136].

Spiromastilactone D was isolated from the deep-sea fungus *Spiromastix* sp. and was able to inhibit influenza virus A/WSN/33 (H1N1) with an IC_50_ value of 6.0 µM, probably by disrupting the interaction between HA and host sialic acid receptors [137].

## 5. Bacterial Extracts and Compounds with Anti-Influenza Properties

Like fungi, bacteria represent a well-known source for the discovery of anti-infectives. However, the majority of research articles are dedicated to the field of antibiotics research. In the following paragraph, we summarize the current knowledge on the antiviral activity of bacterial extracts and compounds against influenza viruses.

### 5.1. Bacterial Extract Screenings

Bacterial secondary metabolites comprise a plethora of bioactive compounds. To keep the complexity of the whole repertoire of compounds in a fermentation broth, the latter was used for screenings. In a study by Bandoro et al., the antiviral effect of 13 heat-inactivated fermentation broths (Bacteroidota: *Bacteroides fragilis*, *B. ovatus*, *B. vulgatus*, *B. thetaiotaomicron*, *Parabacteroides distastonis*; Verrucomicrobiota: *Akkermanisia muciniphilia*; Pseudomonadota: *Escherichia coli* BC 15 and K-12, *Pseudomonas aeruginosa*, *Salmonella enterica*; *Clostridium scindens*, *Eubacterium rectale*, *Ruminococcus obeum*) on various influenza strains was investigated [138]. Only two bacterial strains, *Bacteroides ovatus* and *Akkermansia muciniphilia* had no significant effect on the number of infected cells. Antiviral effects of the other strains were attributed to the bacterial lipopolysaccharides (LPS), which typically are present at the surface of Gram-negative bacteria. The authors hypothesize, that these LPSs might compromise the structural integrity of the viral envelope. Virus stability tests in which different strains like A/Puerto Rico/8/1934 (H1N1), A/mallard/Interior Alaska/10BM11415R0/2010 (H3N8), or A/Brisbane/10/2007 (H3N2) were exposed to heat-inactivated bacteria, confirming the hypothesis.

### 5.2. Cyanobacteria

Cyanobacteria have attracted the attention of researchers, particularly due to the antiviral properties of some of their extracts generated using organic solvents [139].

For example, the crude aqueous extract of the species *Microcystis aeruginosa* waterbloom was tested against influenza A/WSN/33 (H1N1) revealing a 50% inhibitory concentration of 11.0 µg/mL [140]. After subsequent fractionation, the aqueous phase revealed an EC_50_ value of 21.0 µg/mL and the basic compound fraction of 47.0 µg/mL. The inhibition of the proteolytic activity of host proteases is suspected to be the mode of action.

**Figure 8:**
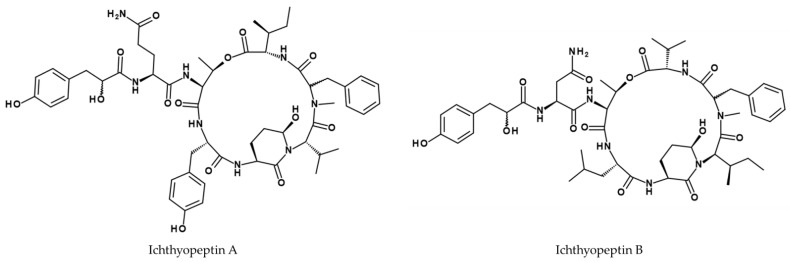
Structures of ichthyopeptin A and B [141].

A fraction containing both small oligopeptides ichthyopeptin A and B (see Figure 8) from *Microcystis ichthyoblabe* strain BM Mi/13 exhibited antiviral effects against influenza virus A/WSN/33 (H1N1) with an IC_50_ value of 12.5 µg/mL [141].

Cyanovirin-N is a lectin produced by the cyanobacterium *Nostoc ellipsosporum* and was originally identified as HIV inhibitor. Furthermore, it inhibits the replication of other enveloped viruses such as influenza as well. This carbohydrate-binding protein targets the high-mannose glycans on the HA [142]. With EC_50_ values ranging from 0.36 to 45.45 nM it is highly active against different IAV strains including the H3N2 subtypes A/Sydney/05/97 and A/Memphis/8/99, the H1N1 subtype A/Beijing/262/95, and the IBV strains B/Hong Kong/5/72, and B/Yamanashi/166/98. Interestingly, the laboratory adapted H1N1 strains A/NWS/33 and A/Puerto Rico/8/34 were significantly less affected with EC_50_ values of ~1 µM. Cyanovirin-N was also tested in a mouse- and ferret-infection model. Early intranasal administration reduced pneumonitis and mortality in mice and in ferrets and a reduction of nasal virus titers was observed [143]. Additionally, cyanovirin-N was used as a probe to study changes in HA of H1N1. Mutations leading to amino acid changes in the binding sites for cyanovirin-N like N94aD resulted in decreased binding and thereby resistance to the compound [144].

*Nostoc flagelliforme* also produces an acidic polysaccharide fraction named nostoflan, which shows activity against the strain A/NWS/33 with an IC_50_ of 96 µg/mL, when added to the media subsequently after the viral infection of the MDCK cells [145]. The strong effect is probably based on the inhibition of interaction between the virus and the host cell.

### 5.3. Pseudomonadota

Other lectins have been isolated from the genus *Pseudomonas*, and have been expressed as recombinant proteins in *E. coli*, namely PFL, PML, and PTL from *Pseudomonas fluorescens, Pseudomonas mandelii, and Pseudomonas taiwanensis* [146]. In plaque assays, a concentration of 0.1 µM of the lectins showed antiviral activity towards H1N1 subtypes A/FM/1/47, A/Bangkok/10/83, A/Beijing/262/95, and A/Oita/OU1P3-3/09, H3N2 subtypes A/Udorn/72 and A/Aichi/2/68, as well as B/Ibaragi/2/85 after pre-incubation with the virus preparations. In the cases of A/Oita/OU1P3-3/09 and A/Udorn/72 even 50% inhibition at around 1 nM were observed with this experimental setup. Like cyanovirin-N, these lectins target viral envelope glycoproteins and hinder viral propagation by blocking interactions with the cells.

*Lactobacteria* and their metabolites are well known for their beneficial effects on human and animal health in general. Their presence, e.g., in the natural acid mantle of human skin or within the gastro-intestinal tract provides a barrier against other bacteria and viruses, which especially accounts for the early stages of defense of the host organism. Similar protective properties have been observed in fermented nutritional products. Various species of *Lactobacillus*, *Streptococcus*, *Enterococcus*, and selected *Bacteriocins* have therefore been investigated for their effect on influenza virus infection [147,148,149].

Nagai et al. demonstrated exopolysaccharides (EPS) of microbial origin to be one component causing enhanced resilience against the mouse-adapted influenza virus A/Puerto Rico/8/34 (H1N1) in BALB/c mice [147]. Infected mice were fed either on a diet supplemented with *L. delbrueckii* ssp. *bulgaricus* OLL1073R-1 and *S. thermophilus* OLS3059 fermented yogurt, or on a diet supplemented with EPS, isolated from OLL1073R-1 culture supernatant. The overall mortality was lower in mice on the supplemented diets. The authors also measured antibody titers (IgA and IgG_1_) and natural killer (NK) cell activity, which all increased in the mice on the yoghurt- or EPS-diet. Finally, neutral EPS (nEPS) was compared to acidic EPS (aEPS). aEPS emerged to protect the mice from influenza while nEPS had no significant effect.

The effectiveness of a heat-killed *L. casei* strain (DK128) was tested by intranasal administration in BALB/c mice infected with the H3N2 influenza virus A/Philippines/2/1982 [149]. Higher doses of DK128 reduced or even eliminated the weight loss caused by the virus infection, concomitant with the depletion of inflammatory cytokines (IL-6 and TNF-α) and virus titers in lung samples and bronchoalveolar lavage fluid.

### 5.4. Actinomycetota

**Figure 9:**
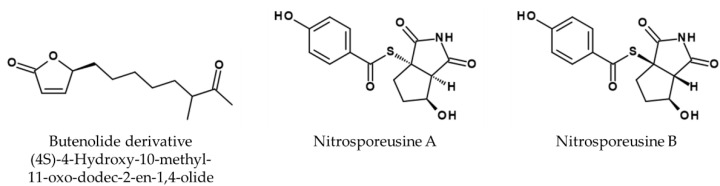
Structure of butenolide derivative ((4*S*)-4-Hydroxy-10-methyl-11-oxo-dodec-2-en-1,4-olide) [151] and nitrosporeusine A and B [153].

The antiviral properties of Actinomycetota have recently been reviewed by Yin et al. [150]. *Streptomyces* sp. Smu03, extracted from the feces of *Elephas maximus*, was shown to produce an antiviral butenolide derivative, namely (4*S*)-4-Hydroxy-10-methyl-11-oxo-dodec-2-en-1,4-olide (see Figure 9), which presumably blocks viral entry into host cells by interfering with the fusogenic process of HA [151]. The following IC_50_ values in µM for A/Aichi/2/68 (H3N2) of 33.84, the mice adapted strain A/FM/1/47 (H1N1) 27.20, A/Puerto Rico/8/34 (H1N1) 1.29, an oseltamivir-resistant variant of A/Puerto Rico/8/34 (H1N1, with NA-H274Y mutation) 16.05, the clinical isolates (H3 subtypes) 690 24.30 and 699 20.46 and a not further-characterized IBV strain 52.12 were reported, respectively.

An analysis of extracts of the extremophile *Actinomycetes* K-192, K-340, K-362, K-522, and K-525, originating from several ecosystems in Kazakhstan, including swamp, saline soils of a clay desert and sor solonchak in steppe and steppe pinewood, revealed their ability to inhibit several influenza virus strains (A/Almaty/8/98 (H3N2), A/Vladivostok/2/09 (H1N1), A/tern/South Africa/1/61 (H5N3) and A/FPV/36/1 (H7N1)) [152]. In the case of H1N1, K-192, K-340, K-522, K-362, and K-525 were shown to inhibit hemagglutination, whereas K-192-1 N, K-192-2 N, and K-192-2S were shown to suppress NA activity of all tested influenza strains. Furthermore, K-362-2 N, K-525-2 N and K525-2S were able to suppress the NA activity of the epidemic strain A/Almaty/8/98 (H3N2), while K-362-1S even inhibited neuraminidase activity of two strains resistant to commercial inhibitors (A/Vladivostok/2/09 (H1N1), A/tern/South Africa/1/61 (H5N3)).

*Streptomyces nitrosporeus* CQT14-24 isolated from the arctic Chukchi Sea produces the alkaloids nitrosporeusines A and B (see Figure 9). The inhibitory rates of nitrosporeusine A and B using 50 µM are 18.6% and 30.9%, respectively, whereas oseltamivir phosphate inhibited 54.0% in the same experiment. The latter inhibits strain A/WSN/33 (H1N1) with an EC_50_ value of 112.7 µM in a viral plaque assay [153].

**Figure 10:**
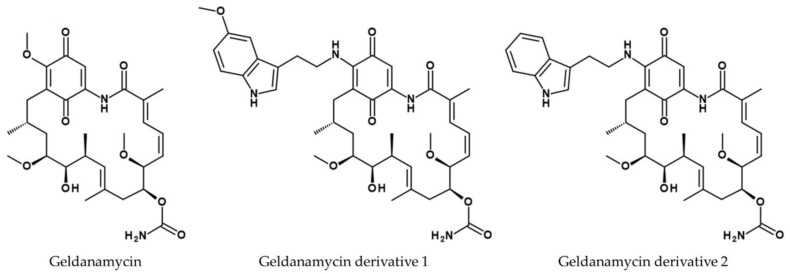
Structures of geldanamycin, 17-(tryptamine)-17-demethoxy geldanamycin (derivative 1) and 17-(5′-methoxytryptamine)-17-demethoxygeldanamycin (derivative 2) [157].

Virus replication can be interrupted by blocking protein synthesis, which is the mechanism of action of geldanamycin (see Figure 10) [154], which was found to be produced by *Streptomyces hygroscopicus var. geldanus var. nova* [155] and *Streptomyces zerumbet* [156]. This heat shock protein (HSP) 90 inhibitor is only sparingly soluble in water and is also hepatotoxic [157]. However, replacing a methyl ether group with different nucleophiles results in 17-(tryptamine)-17-demethoxy-geldanamycin (derivative 1, see Figure 10) and 17-(5′-methoxytryptamine)-17-demethoxy-geldanamycin (derivative 2, see Figure 10), which show lower toxicity and higher solubility in water. The IC_50_ value for cytotoxicity of geldanamycin was 128.20 µM in LLC-MK2 cells and 96.40 µM in Vero cells, but this increased to more than 290 µM in both cell lines for derivatives 1 and 2. The compounds inhibited viral propagation of A/free-grazing duck/Nakhon Pathom/1/2017 (H5N2) at concentrations of 21.7 µM for geldanamycin and 17.39 and 18.15 µM for the derivatives 1 and 2, respectively. The hemagglutination inhibition titer for geldanamycin was not determined, whereas the titer for both derivatives was 1:50.

Seven fluvirucins (A_1_, A_2_, B_1_, B_2_, B_3_, B_4_, and B_5_) were purified from extracts of five unidentified *Actinomycetes* species Q464-31, R869-90, R359-5, R516-16, and L407-5, revealing the following ID_50_ values for compound A1 10.72 µM, A2 9.37 µM, B1 5.19 µM, B2 21.67 µM, B3 10.07 µM, B4 32.78 µM, and B5 > 82.78 µM, respectively, against an unspecified IAV [158].

**Figure 11:**
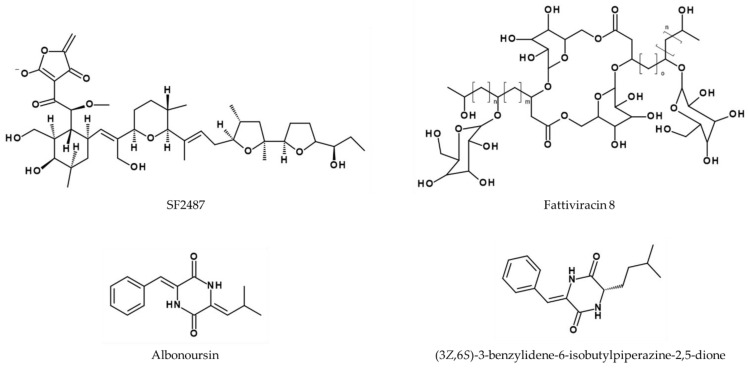
Structure of SF2487 [159] and of fattiviracin 8 with n = 5, m = 13, and o = 11 [160], albonoursin, and (3*Z*,6*S*)-3-benzylidene-6-isobutylpiperazine-2,5-dione [163].

*Actinomadura* sp. SF2487 produces an antiviral compound (also called SF2487, see Figure 11) with activity against A/Puerto Rico/8/34 [159]. A dose-dependency was observed for SF2487 from 0 to 1.32 µM, which was shown by an increased rate of inhibition in a viral plaque assay.

*Streptomyces microflavus* 2445 produces at least 13 glycolipid derivatives of the so-called fattiviracins (FV-1–FV-13). Two compounds exhibiting anti-influenza activity are fattiviracin A1 and fattiviracin 8 (FV-8), which consists of four glucose and two trihydroxy fatty acid residues (see Figure 11). FV-8 has no cytotoxic effects on MDCK cells and protects them against H1N1 (not further specified) and IBV B/Lee/40 with EC_50_ values of 1.34 and 2.33 µM, respectively [160,161,162]. Similarly, fattiviracin A1 had an antiviral EC_50_ of 1.34 µM against H1N1 (not further specified). Interestingly, fattiviracins show a broad antiviral effect, with virus-related differences depending on their pharmacodynamic properties. The supposed mechanism is their integration into membranes due to their amphipathicity with hydrophilic sugar and lipophilic lipid structures, thus modulating membrane fluidity, which results in blocking of fusion-pore-formation [161].

The fermentation broth of *Streptomyces* sp. FXJ7.328 was shown to contain the diketopiperazine derivative (3*Z*,6*Z*)-3-(4-hydroxybenzylidene)-6-isobutylidenepiperazine-2,5-dione. Its activity against A/Puerto Rico/8/34 (H1N1) was compared to the known diketopiperazine derivatives (3*Z*,6*S*)-3-benzylidene-6-isobutylpiperazine-2,5-dione, and albonoursin (Figure 11) in MDCK cells, revealing IC_50_ values of 41.5 ± 4.5 µM for the new compound compared to 28.9 ± 2.2 µM for (3*Z*,6*S*)-3-benzylidene-6-isobutylpiperazine-2,5-dione and 6.6 ± 1.5 µM for albonoursin. The antiviral activity of these compounds was conferred by specific chemical moieties, with *Z*-dehydro-Phe and Leu or *Z*-dehydro-Leu groups showing the most potent effects. *E*-isomerization, *N*-methylation, and the hydroxylation of dehydro-Phe or dehydro-Leu was associated with lower activity. The same effect was observed when dehydro-Phe and dehydro-Tyr were replaced with dehydro-His and Leu, or with dehydro-Leu with Ile [163].

*S. lavendulae* DSM 41290 culture filtrate and two of its active components, one of them being Ehrlichin, were tested against A/Puerto Rico/8/34 (H1N1) and B/Lee/40 in embryonated chicken eggs in vitro, and in some cases also in vivo [164]. The protective and therapeutic impact was investigated by measuring HA levels in the embryo’s blood, confirming hemagglutination in embryos infected with the influenza B strain.

Besides favipiravir, additional natural nucleotide and nucleoside analogs with activity against influenza have been described and were recently reviewed [165,166,167]. For example, broad-spectrum antiviral activity, including influenza, was found for the nucleosides formycin A and B derived from *Streptomyces candidus* [168,169]. For formycin A an IC_50_ value of 37.3 ± 21.5 nM was determined against A/WSN/33 (H1N1) in MDCK cells. Its thiomethylated derivative possesses IC_50_ values of 34.1 ± 27.4 nM against A/WSN/33 (H1N1), 43.8 ± 27.7 nM against A/equine/2/Miami/63 (H3N8), and 37.9 ± 26.6 nM against B/Lee/40 in MDCK cells [170]. However, the known teratogenic potential of some nucleotide and nucleoside analogs suggests that formycin also harbors a potential risk, which could be overcome by further molecular modifications.

One particularly promising candidate is DAS181 (Fludase^®^). This antiviral protein features the catalytic domain of *Actinomyces viscosus* sialidase (AvCD) combined with the GAG-binding sequence of human protein amphiregulin (AR) as an anchoring domain. DAS181 was shown to remove α-(2,3)- and α-(2,6)-linked sialic acid thus preventing the virus from entering the host-cell. DAS181 was expressed as a fusion protein in *E. coli* and tested in various cell lines (MDCK, A549, Caco-2) and human airway epithelium cultures to confirm tolerance. Viral inhibition assays in MDCK cells allowed the calculation of EC_50_ and EC_90_ values against various influenza virus strains [171]. The following EC_50_ values in nM were reported: H1N1: A/Puerto Rico/8/34: 0.8; A/WSN/33: 0.4; A/Weiss/43: 0.9; A/Denver/1/57: 0.5; H2N2: A/Japan/305/57: 0.3; H3N2: A/Victoria/504/2000: 0.4; A/Hong Kong/8/68: 0.1; A/Port Chalmers/1/73: 0.05; A/Victoria/3/75: 0.1; H9N2: A/turkey/Wisconsin/66: 0.4 and the IBV subtypes B/Lee/40: 0.3; B/Maryland/1/59: 0.04. For clinical isolates, the following EC_50_ values were reported: H1N1: A/Hong Kong/2637/2004: 0.2; A/Hong Kong/2765/2004: 1.2; H3N2: A/Singapore/35/2004: 0.6; A/Canada/600/2004: 0.1; A/Texas/05/2004: 0.2; and the IBV subtype B/Peru/1960/2004: 0.2. Because some of the EC_50_ values were within the error range of the experimental method, further in vivo experiments were carried out by intranasal administration in mice, confirming the efficacy of a 25 unit/treatment dose. The activity of closely related analog DAS178 was also tested in ferrets, as a model of human influenza infections, and the results were similar to those reported in mice [171].

DAS181 has also been tested against A/Vietnam/1203/2004 (H5N1) in BALB/c mice [172]. Pretreatment conferred strong protection against intranasal infection, but post-infection treatment was also therapeutically efficacious. Mice were challenged by intranasal inoculation with either 3 MLD_50_ or 1.5 MLD_50_, while application of DAS181 started one day before infection and continued for 7 days. DAS181 was administered at doses of 1 mg/kg/d, 0.7 mg/kg/d (twice a day 0.3 mg/kg), or 0.3 mg/kg/d. In the group infected with 3 MLD_50_, the survival and infection rates were as follows: 1 mg/kg/d = 100% survival with 33% infected, 0.7 mg/kg/d = 69% survival with 88% infected, and 0.3 mg/kg/d = 40% survival with 87% infected. In the group infected with 1.5 MLD_50_, the survival and infection rates were as follows: 1 mg/kg/d = 100% survival with 29% infected, 0.7 mg/kg/d = 94% survival with 81% infected, and 0.3 mg/kg/d = 47% survival with 71% infected mice. The virus titers in the lungs of the mice were reduced by at least 10,000-fold depending on the dose of DAS181, and no virus was detected in the brains of any mice treated with DAS181. To underline the efficacy of DAS181, the presence of antibodies was investigated. The group of mice treated with 0.3 and 0.7 mg/kg/d produced H5-specific antibodies while the group treated with 1 mg/kg/d did not. This proves that this group was not productively infected with the influenza virus. In addition, the efficacy as post-infection therapeutic was investigated with mice infection with 3 MLD_50_ and intranasal administration of DAS181 at 1 mg/kg/d starting 24, 48, or 72 h post infection. Results showed, the more time passed by between infection and treatment, the less the protective effects of DAS181 could develop (70% survival rate at 24 h and 35% at 72 h post infection) [172]. Based on these results, a phase II clinical trial of DAS181 was initiated in 2012 in New York. This trial, including patients infected with influenza B, influenza A 2009 pandemic H1N1 and seasonal H3N2 strains could confirm the antiviral effect of DAS181 [173]. Furthermore, in a different study, DAS181 effectively inhibited replication of subtype H7N9 (strains A/Shanghai/1/2013 and A/Taiwan/1/2013) [174]. DAS181 was also tested against parainfluenza viruses. In 2016 it was experimentally applied to transplant recipients in hospital in New York who had been diagnosed with parainfluenza viruses [175]. Here, DAS181 also showed positive effects. Due to the promising results of the phase II clinical trial, a phase III (NCT03808922) was initiated and is currently ongoing.

## 6. Conclusions and Future Directions

Despite being well studied, influenza remains a continuous threat to global health. Besides seasonal outbreaks with varying mortality rates and a huge economic burden, influenza poses a significant pandemic threat, due to its vast animal reservoir and genetic flexibility. However, the treatment options are still limited. So far, only M2 channel blockers and neuraminidase inhibitors are approved in most countries. Both classes of inhibitors suffer from a lack of efficacy and are susceptible to the development of resistances. Additional drugs are approved in some countries but often suffer from similar drawbacks or are limited in use. Therefore, novel antiviral therapeutics against influenza infections are urgently needed.

Over the past decades, considerable efforts have been made to develop novel therapeutics to combat influenza virus infections. The enormous diversity and structural complexity make natural products a promising starting point for the exploration and identification of novel compounds exhibiting activity against influenza viruses. In this review we presented the current research on natural products like toxins and AMPs as well as compounds and extracts isolated from fungi and bacteria with anti-influenza activity. Most of these compounds are at a very early stage of antiviral research. Due to several reasons, a comparison of the compounds in terms of their suitability as antiviral drug is difficult. Firstly, the methods used to characterize the compounds vary widely. For example, in many studies cell culture assays are performed in canine MDCK cells or in Vero cells; human cell lines are seldom employed. Additionally, the readout used to assess inhibition of virus replication differs. While many groups determine viral titers via plaque assay, others use the hemagglutination assay or the quantification of the virus-induced cytopathic effect to determine antiviral activity. Therefore, defining standardized methods to assess antiviral efficacy would be helpful for a better comparison. Secondly, for many compounds the mechanism of action is not identified. However, the understanding by which the compounds act is crucial for the drug development process. Thirdly, for most compounds, no data on resistance development are available. Even at an early stage it is possible to determine the potential of resistance development in serial passage experiments in presence of the compound. Only for a few compounds like DAS181 or cyanovirin-N in vivo data are available and only one namely DAS181 is currently tested in a phase III clinical trial, however only against parainfluenza infections.

Considering the high costs of in-depth characterization, for example in in vivo studies, close cooperation between academic and public-funded organizations and industry e.g., in public private-partnership or consortia, are key for the development of novel anti-influenza compounds. This strategy has already been employed very successfully for other infectious diseases. For example, fexinidazole was developed by Hoechst (now Sanofi) in 1978 for the treatment of leishmaniosis but its development was halted at an early stage. In 2005 the non-profit initiative Drugs for Neglected Diseases (DNDi) and the Swiss Tropical and Public Health Institute rediscovered fexinidazole in a huge screening for anti-parasitic activity. In a partnership between DNDi and Sanofi, the compound was finally developed further and is now used to treat sleeping sickness in endemic countries.

Additionally, besides the mono-therapy with one compound a combination therapy should be considered. In case of other viral infections like HIV, a combination of up to four drugs acting on different viral targets, known as highly active antiretroviral therapy (HAART), has proved to be beneficial for an efficient therapy compared to the treatment with a single compound in terms of synergistic effects and reduction of resistances. For influenza, the combination of NA inhibitors with adamantanes, favipiravir, or ribavirin was already successfully tested in cell culture and showed increased efficacy and prolonged survival of infected mice compared to the monotherapy [176,177,178,179,180].

With our summary on the testing and characterization of natural products as antivirals, we intend to provide novel ideas and suggestions for the future development of novel anti-influenza drugs either as mono- or in combination therapy.

## Nomenclature

- EC_50_—Effective concentration of a compound where the infectivity is reduced by half
- IC_50_—Inhibitory concentration of an inhibitor where the response is reduced by half
- ID_50_—Virus induced cytopathic effect of an inhibitor is reduced by half
- MLD_50_—Murine lethal dose of a substance needed to kill half the members of the tested population
- TD_50_—Median toxic dose of a drug at which toxicity occurs in 50% of the cases
